# Computational screening of natural inhibitors against *Plasmodium falciparum* kinases: Toward novel antimalarial therapies

**DOI:** 10.1371/journal.pone.0339317

**Published:** 2026-01-13

**Authors:** Muharib Alruwaili, Sonia Younas, Abozer Y. Elderdery, Intisar Alruwaili, Jeremy Millis, Muhammad Umer Khan, Hasan Ejaz

**Affiliations:** 1 Department of Clinical Laboratory Sciences, College of Applied Medical Sciences, Jouf University, Sakaka, Saudi Arabia; 2 Centre for Immunology and Infection (C2i), Hong Kong Science and Technology Park, Hong Kong, SAR China; 3 HKU-Pasteur Research Pole, School of Public Health, LKS Faculty of Medicine, The University of Hong Kong, Hong Kong, SAR China; 4 School of Medicine, Pharmacy and Biomedical Sciences, University of Portsmouth, Portsmouth, United Kingdom; 5 Institute of Molecular Biology and Biotechnology, The University of Lahore, Lahore, Pakistan; Kwara State University, NIGERIA

## Abstract

An important worldwide problem is the resistance of *Plasmodium falciparum* to practically all antimalarial medications. Therefore, new treatment approaches are urgently needed. The development of antimalarial medications frequently involves two important therapeutic targets: casein kinase 2 (CK2) and cGMP-dependent protein kinase (PKG). To identify naturally occurring chemicals that could be used as antimalarial medications to combat multidrug-resistant *P. falciparum*, we used a multi-targeted *in silico* strategy in this study. The top 20 compounds, including the reference drug RY-1–65, were selected after pharmacophore-based virtual screening of naturally produced compounds. These compounds were subsequently docked onto both target proteins using Maestro (Schrödinger 2020−3). The best-scoring compounds against PKG and CK2 were Ligand-9 (−7.490 kcal/mol) and Ligand-13 (−11.468 kcal/mol), respectively. These lead compounds may be useful as therapeutic targets based on an assessment of their pharmacological, toxicological, and bioactivity characteristics. Furthermore, Ligand-13’s strong reactivity and stability were demonstrated by density functional theory analysis, and these findings were confirmed by molecular dynamics simulations and binding free energy MMGBSA calculations. These results imply that Ligand-13 may be a promising antimalarial medication.

## 1. Introduction

An estimated 249 million clinical cases of malaria and 608,000 deaths due to the disease were reported globally in 2022. The World Health Organization’s most recent World Malaria 2024 report estimates that there were 597 000 malaria fatalities and 263 million cases globally in 2023. This translates to almost the same number of deaths and approximately 11 million more cases in 2023 than in 2022. In the WHO African Region, where many at-risk individuals still do not have access to the services necessary to prevent, detect, and treat the disease, approximately 95% of the deaths occurred [1]. Malaria is a vector-borne disease caused by a protozoan organism in the genus *Plasmodium* [2]. *P. falciparum* is by far the most virulent as well as the most prevalent species worldwide [3]. It is recognized as the deadliest malarial species due to the high number of deaths associated with it [4]. In most malaria-endemic nations, the World Health Organization recommends artemisinin-based combination therapy (ACT) as the first-line treatment for uncomplicated malaria [5,6]. The control of malaria is seriously threatened by the rise of antimalarial medication resistance in *P. falciparum*. Artemisinin resistance has developed and is mostly found in Southeast Asia, with few instances in Africa [7]. It is interesting to note that this drug resistance has spread to include other medications taken in combination with ACT. Moreover, resistance to commonly used treatment medications has also resulted from multidrug-resistant *Plasmodium* strains [8]. Malaria’s prevalence and drug resistance highlight the need to address enduring treatment hurdles despite efforts to eradicate the disease.

Multi-targeted therapy is necessary to eradicate *P. falciparum* because of its intricate life cycle [9]. Casein kinase 2 (CK2) and cGMP-dependent protein kinase (PKG) are two important kinase proteins used for this purpose. In *P. falciparum*, PKG is the primary effector of cGMP signaling. Additionally, in the erythrocytic and pre-erythrocytic embryonic stages of P. falciparum, it controls both the sexual and asexual cycles [10]. It has also been recognized as a possible target for antimalarial drugs in earlier studies [11,12]. CK2 in *P. falciparum* comprises three chains: two β-chains (B and C) for regulatory roles and one α-chain (A) for catalytic activity. The nucleus contains the alpha chain of CK2 (CK2α), which is specifically implicated in chromatin phosphorylation [13]. Furthermore, CK2 plays a role in the development of both the sexual and asexual blood stages [14].

The identification and preliminary characterization of a novel imidazole-based chemotype, RY-1–165, have been documented in previous studies. This chemotype exhibits promising cellular activity, good *in vitro* PfPKG inhibition, a correlation between *in vitro* enzymatic activity and efficacy, no structural alerts linked to genotoxicity, and no hERG problems, similar to other chemotypes [15]. The cyclopropyl group of compound 14a (RY-1–165) resulted in a three-fold increase in potency (IC_50_ = 100 nM, E_50_ = 7.6 μM) as a PfPKG inhibitor.[16].

The antimalarial drug of the future should ideally be a single-dose, cost-effective therapy capable of addressing parasite resistance. These conditions could be fulfilled through novel, easily synthesized natural compounds or their derivatives with selective antimalarial activity. Natural products such as chloroquine from the South American cinchona trees and artemisinin from the Chinese *Artemisia annua* are effective antimalarial drugs [17,18]. Despite resistance to these drugs, there is a need for further exploration of natural biotherapeutics.

*In silico* methods, which save time and money while enabling the assessment of novel molecule properties, such as toxicity and efficiency, before synthesis, can accelerate drug development [19]. Molecular docking and molecular dynamics (MD) simulations are two essential methods in molecular biology and computer-aided drug design. Molecular docking is frequently used to predict the binding orientation of therapeutic candidates to their protein targets to estimate the affinity and activity of small molecules [20]. In *P. falciparum*, PKG is the primary effector of cGMP signaling. Previous studies have identified PKG as a target, shedding light on the potential of inhibitors to prevent malaria. For instance, the interaction and stability of PKG-inhibitor complexes have been examined using docking and molecular dynamics simulations [21].

Therefore, this study employed an extensive *in silico* approach to explore and identify novel naturally derived compounds that can act as inhibitors against *P. falciparum*’s PKG and CK2 proteins to selectively target the malarial life cycle to safely and cost-effectively overcome antimalarial resistance.

## 2. Methodology

**Fig 1:**
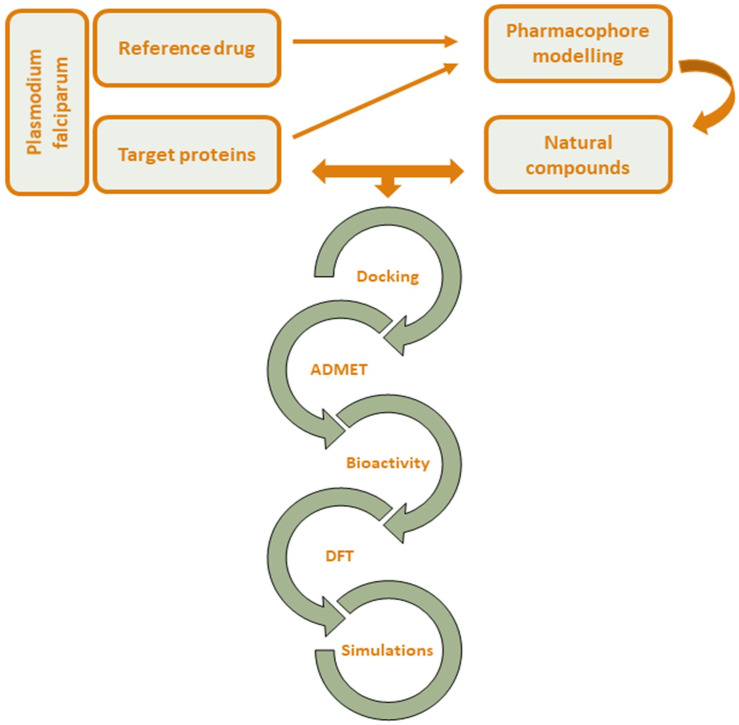
The workflow of the *in silico*-based methodology.

The workflow of this *in silico*-based study is outlined in **Fig 1**.

### 2.1. Target proteins

The target proteins were retrieved in Protein Data Bank (PDB) format from protein data bank (PDB) database [22]. Target-1, PKG (PDB ID: 8EM8), consisted of 857 amino acids and a molecular weight of 98.63 kDa [16]. Target-2, CK2 (PDB ID: 5XVU), consisted of 996 residues, a molecular weight of 121.58 kDa, and 3 chains (A, B, C) [23].

### 2.2. Reference inhibitor

The reference inhibitor for pharmacophore-based virtual screening (PBVS) of naturally derived compounds for both target proteins was RY-1–65 (PDB ID: 8EM8), a trisubstituted derivative of the antimalarial drug imidazole [16,15]. It was retrieved via the PubChem (CID: 164628676) database in SDF format.

### 2.3. Pharmacophore-based virtual screening

The PBVS was performed via Pharmit [24] using the crystal structures of the target proteins and the selected reference inhibitor. Pharmit ZINC Natural library, consisting of 1,441,177 conformers of 116,363 molecules, was searched and root mean square deviation (RMSD)-based top 20 naturally derived compounds were retrieved from the ZINC database [25] for docking analysis.

### 2.4. Molecular docking

The 2D structures of each chosen compound were manually designed using ChemDraw Professional 16.0. The 2D structures were then converted to 3D using Chem3D 16.0 [26], and the resulting files were saved as sdf. The Chem3D Gaussian interface was used to minimize energy. The SDF files of each ligand underwent ligand processing to assign the proper bond order [27].

The protein data library provides the 3D crystal structures of the target proteins for download. Protein preparation was carried out using Mgl tools 1.5.7 and Biovia Discovery Studio software in accordance with the usual procedure. Cofactors and water molecules were removed. After removing the previously joined ligands, AutoPrep was used to add polar hydrogens and Kollman’s charges to create the protein [28].

The “receptor cavity method” was employed to predict the binding sites of receptor proteins using BIOVIA Discovery Studio 2021. The SDB-Site module of the BIOVIA Discovery Studio program enabled the identification and description of protein structural binding sites. This process utilized the inhibitory properties of residues present in the binding sites with center-x, y, and z values [27].

The molecular docking was performed using AutoDock Vina 1.5.7 [29]. Following preparation, the receptor and ligand structures were saved in pdbqt format and transferred to the Vina folder. Vina’s AutoDock application was launched using the Command Prompt (CMD). The program command executed was “vina --config conf.txt --log log.txt” [30]. This method uses a grid-based model of protein-ligand potential interactions to calculate the binding affinity. Soft-core potentials, which are employed by Vina tools, are helpful in generating a range of random conformations of tiny organics and macromolecules inside the active area of the target protein. After docking the ligands to the proteins, the relative intensity of their interactions was assessed to identify possible treatment options. The test ligands were docked after the co-crystallized ligand was redocked to ascertain its binding affinity for the target protein [31]. Lastly, the docking results were displayed in terms of docking score, and top-identified compounds were visualized using PyMOL (version 2.4.0) for a 3D view [32] and BIOVIA Discovery Studio 2024 (v24.1.0.23298) for 2D interaction [33,34].

### 2.5. ADMET analysis

To predict the effectiveness and safety of top-identified compounds, their ADMET (absorption, distribution, metabolism, excretion, and toxicity) properties were determined. A web server, SwissADME [35], was used to predict the ADME (absorption, distribution, metabolism, and excretion) properties of the compounds. The top hits for both targets were compared with reference drugs. Toxicity analysis was performed using StopTox [36], a web server. The Simplified Molecular Input Line Entry System (SMILES) string of the compounds was incorporated as input files to run predictions in both web servers.

### 2.5. Bioactivity prediction

For ligand-based target prediction, Swiss Target Prediction [37] was applied, which determined the most likely protein targets. The top-identified compounds for both targets, along with the reference drug, were subjected to bioactivity prediction.

### 2.6. Density function theory analysis

The Gaussian 09 program [38] was employed to perform density function theory (DFT) calculations for the reference drug and the top-identified compounds for each target, followed by visualizations of molecular electrostatic potentials (MEPs). For conductor-like polarizable continuum model (CPCM) optimization and frequency calculations for physiological phase, the B3LYP approach was selected. In addition, the HOMO–LUMO energy gap was optimized using Gaussian 09, with VESTA (version 3) applied to visualize their models for enhanced computational clarity [39,40].

### 2.7. Molecular dynamics simulations

Ligand-**13** and reference drug in complex with Target-**2** (CK2α) underwent molecular dynamics (MD) simulations with Amber17 to gain more knowledge about the stability of the chemical structure of the ligand when bound to the target protein. Initially, the process was facilitated by assigning partial atomic charges to Ligand-**13** and the reference drug using the Antechamber module of Amber. Then, both compounds and the target protein (CK2α) were subjected to the Leap module, in which missing hydrogen atoms were added, the system was neutralized, and the complexes were solvated followed by the generation of necessary parameters and coordinate files for subsequent simulations. Then, for characterization of the dynamic properties of the system, the ff14SB force field was applied to the protein, while the generalized Amber force field (GAFF) was used for Ligand-**13** and the reference drug. Two chloride ions were added to neutralize the protonated protein, and the complex was solvated in an octahedral box with TIP3P water, maintaining a margin distance of 10.0 Å. Finally, the necessary coordinate and parameter files were generated for the simulation, and the solvated complex was stored in the PDB format.

The complexes were reduced during the simulations to eliminate steric conflicts. Following minimization, the system was progressively heated from 0 to 300 K. To further stabilize the system, it underwent another equilibrium stage at 300 K. Equilibrium was established using Langevin dynamics with a force constant of 10 kcal/(mol Å²) and a collision frequency of 1 ps ⁻ ¹. After stabilization, the NPT ensemble was used to run 100 ns MD simulations at 300 K and 1 atm [41].

Several parameters, including RMSD, solvent-accessible surface area (SASA), and root mean square fluctuation (RMSF), were calculated at the end of the simulations using the CPPTRAJ module of Amber 17 [41] The radius of gyration was calculated for 100 ns using the method described by Arantes et al [42]. Principal component analysis and dynamic cross-correlation (DCCM) were also performed using the Bio3D package of R by writing a script specifically for these calculations [43–45].

### 2.8. Binding free energy MMGBSA calculations

Using MD shots, the binding free energy was computed to learn more about the energetic values and structure of the complexes. The MMPBSA/MMGBSA modules were used in conjunction with Amber17 to accomplish this [46]. These data were obtained by calculating the final 2 ns of data from 1000 snapshots of both complexes to determine the overall energies of Ligand-**13** and the reference drug in complex with Target-**2** (CK2α) using the following equation [47]:


$$\Delta {\text{G}}_{\text{bind}}=\;\Delta G_{\text{complex}}\_(\Delta G_{\text{protein}}+\Delta G_{\text{ligand}})$$ (1)

The molecular mechanics energy (E_mm_) was calculated using the following equation:


$${\text{E}}_{\text{mm}}=\Delta E_{\text{ref}}+\Delta E_{\text{vdW}}+\Delta E_{\text{ele}}$$ (2)

The fragmentation of E_mm_ was analyzed as van der Waals (vdW) energies, non-bonded electrostatic energies (E_ele_), and solvation-free energy (G_sol_) encompassing both polar and non-polar:


$$\Delta G_{\text{sol}}=\Delta G_{\text{ele},\;\text{solPB}(\text{GB})\;}+\Delta G_{nonpol,\;sol}$$ (3)

## 3. Results

### 3.1. Target proteins

**Fig 2:**
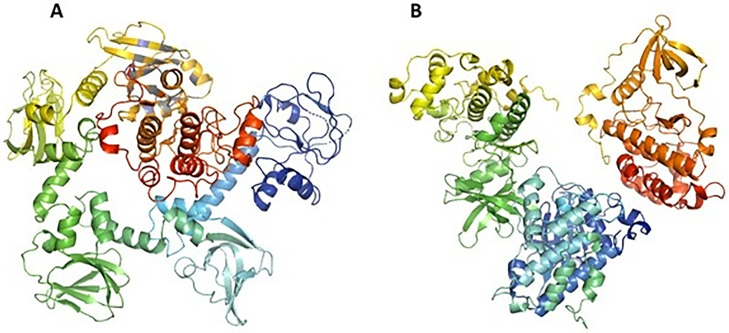
Selected target proteins of *Plasmodium falciparum.* **A**: cGMP-dependent protein kinase PKG. **B**: Protein kinase CK2 catalytic domain.

**Fig 2** shows the 3D structures of the two target proteins after the removal of unnecessary associated structures such as ions and small molecules.

### 3.2. Pharmacophore-based virtual screening

**Fig 3:**
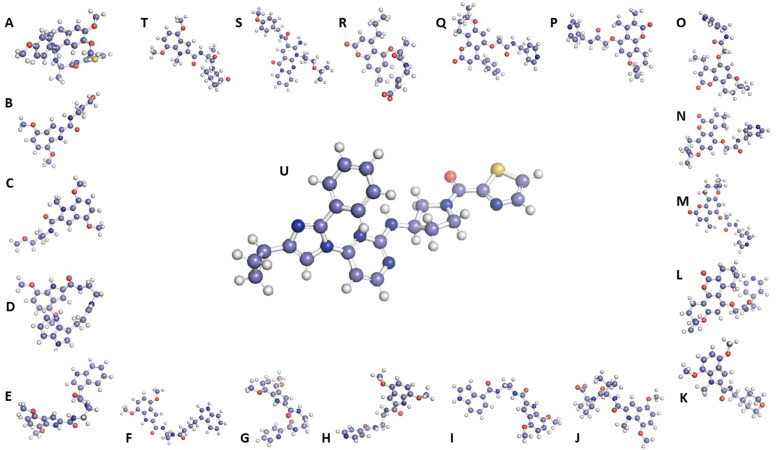
The 3D structures of the reference compound (U) and top 20 pharmacophore-based virtually screened naturally derived compounds (Ligand-1 to Ligand-20 presented chronologically as A to T).

The naturally-derived compounds from the PBVS were sorted based on their RMSD values, and the top 20 compounds with the lowest RMSD values were downloaded (**Table 1**) and visualized along with the reference drug in a ball-and-stick presentation using PyMOL (**Fig 3**).

**Table 1:** Top 20 Pharmit-screened naturally derived compounds arranged in increasing order of root mean square deviation (RMSD) values.

Ligand No.	Name	RMSD	Mass	Molecular formula	SMILES
1	ZINC000080357893	0.549	497	C25H28N4O5S	COc1ccc([C@@H]2c3cc(OC)c(OC)cc3CCN2C(=O)N[C@H](C)C(=O)Nc2nccs2)cc1
2	ZINC000096114719	0.619	278	C14H18N2O4	COc1ccc(OC)c2[nH]c(C(=O)NCCCO)cc12
3	ZINC000072324524	0.628	306	C16H22N2O4	COCCCNC(=O)c1cc2c(OC)ccc(OC)c2n1C
4	ZINC000040312708	0.648	434	C24H26N4O4	COc1ccc(OC)c2[nH]c(C(=O)NCCNC(=O)CCc3c[nH]c4ccccc34)cc12
5	ZINC000040309277	0.648	420	C23H24N4O4	COc1ccc(OC)c2[nH]c(C(=O)NCCNC(=O)Cc3c[nH]c4ccccc34)cc12
6	ZINC000040312547	0.648	449	C25H28N4O4	COc1ccc(OC)c2[nH]c(C(=O)NCCNC(=O)CCCc3c[nH]c4ccccc34)cc12
7	ZINC000040312417	0.649	368	C19H20N4O4	COc1ccc(OC)c2[nH]c(C(=O)NCCNC(=O)c3ccccn3)cc12
8	ZINC000040309843	0.649	368	C19H20N4O4	COc1ccc(OC)c2[nH]c(C(=O)NCCNC(=O)c3ccncc3)cc12
9	ZINC000040312783	0.649	406	C22H22N4O4	COc1ccc(OC)c2[nH]c(C(=O)NCCNC(=O)c3cccc4[nH]ccc34)cc12
10	ZINC00008790317	0.653	430	C22H29N3O6	COc1ccc(OC)c2c1cc(C(=O)N[C@H](C(=O)N1CCC[C@H]1C(=O)O)C(C)C)n2C
11	ZINC00004000361	0.663	404	C20H27N3O6	COc1ccc(OC)c2c1cc(C(=O)N[C@@H](C)C(=O)N[C@H](C(=O)O)C(C)C)n2C
12	ZINC00100757411	0.676	476	C26H29N5O4	COc1ccc(CNC(=O)c2cc3c(=O)n4ccccc4nc3n(CCCOC(C)C)c2 = N)cc1
13	ZINC00002123745	0.680	374	C20H25NO6	CCCc1cc(=O)oc2cc(C)cc(O[C@@H](C)C(=O)NCCCC(=O)O)c12
14	ZINC00008792048	0.688	471	C28H26N2O5	CC1(C)CCc2c(cc(OCC(=O)NCc3ccncc3)c3c(-c4ccccc4)cc(=O)oc23)O1
15	ZINC00004000468	0.689	422	C24H26N2O5	Cc1c(C)c2c(OCC(=O)NCc3ccncc3)cc3c(c2oc1=O)CCC(C)(C)O3
16	ZINC00004000484	0.689	422	C24H26N2O5	CCc1cc(=O)oc2c3c(cc(OCC(=O)NCc4ccncc4)c12)OC(C)(C)CC3
17	ZINC00008791434	0.689	434	C25H26N2O5	CC1(C)CCc2c(cc(OCC(=O)NCc3ccncc3)c3c4c(c(=O)oc23)CCC4)O1
18	ZINC00004000467	0.689	408	C23H24N2O5	Cc1cc(=O)oc2c3c(cc(OCC(=O)NCc4ccncc4)c12)OC(C)(C)CC3
19	ZINC00008792052	0.690	449	C26H28N2O5	CC1(C)CCc2c(cc(OCC(=O)NCc3ccncc3)c3c4c(c(=O)oc23)CCCC4)O1
20	ZINC000072324958	0.695	332	C18H24N2O4	COc1ccc(OC)c2c1cc(C(=O)NCC1CCOCC1)n2C

### 3.3. Molecular docking

The docking results (**Table 2**) showed that the reference drug had a docking score of −9.054 kcal/mol and −7.683 kcal/mol with PKG and CK2, respectively. For PKG, the reference inhibitor showed the best docking score, followed by Ligand-**9** with a score of −7.490 kcal/mol. For CK2, the best docking score was with Ligand-**13**, i.e., −11.468 kcal/mol. Thus, Ligand-**9** and Ligand**-13** were identified as the lead compounds.

**Table 2:** Docking scores of target proteins (PKG and CK2) with the reference drug (PubChem CID: 164628676) and pharmacophore-based virtually screened naturally derived compounds generated by Maestro.

Ligand no.	Name	Docking score (kcal/mol)	
		Target-1	Target-2
Reference drug	PubChem CID: 164628676	−9.054	−7.683
1	ZINC000080357893	−4.373	−2.525
2	ZINC000096114719	−6.114	−6.522
3	ZINC000072324524	−5.055	−4.755
4	ZINC000040312708	−7.121	−7.525
5	ZINC000040309277	−5.445	−5.516
6	ZINC000040312547	−5.293	−7.359
7	ZINC000040312417	−5.906	−3.527
8	ZINC000040309843	−4.843	−6.579
9	ZINC000040312783	−7.490	−6.638
10	ZINC00008790317	−4.847	−5.662
11	ZINC00004000361	−5.209	−8.655
12	ZINC00100757411	−4.484	−5.275
13	ZINC00002123745	−6.193	−11.468
14	ZINC00008792048	−6.611	−8.997
15	ZINC00004000468	−6.732	−9.627
16	ZINC00004000484	−5.494	−10.580
17	ZINC00008791434	−6.862	−8.166
18	ZINC00004000467	−5.675	−8.858
19	ZINC00008792052	−5.210	−6.456
20	ZINC000072324958	−5.019	−5.277

Ligand-**9** and Ligand-**13**, the top compounds identified based on the docking score, were selected for their specific interactions with Target-**1** (PKG) and Target-**2 (CK2)**, respectively. The interactions were visualized on Discovery Studio and PyMol in comparison with the reference drug. All four selected docked complexes exhibited van der Waals, conventional hydrogen bond, and carbon-hydrogen bond interactions, while pi–cation, pi–sulfur, and salt bridges were observed selectively (Supplementary Table S1 in S1 File).

**Fig 4:**
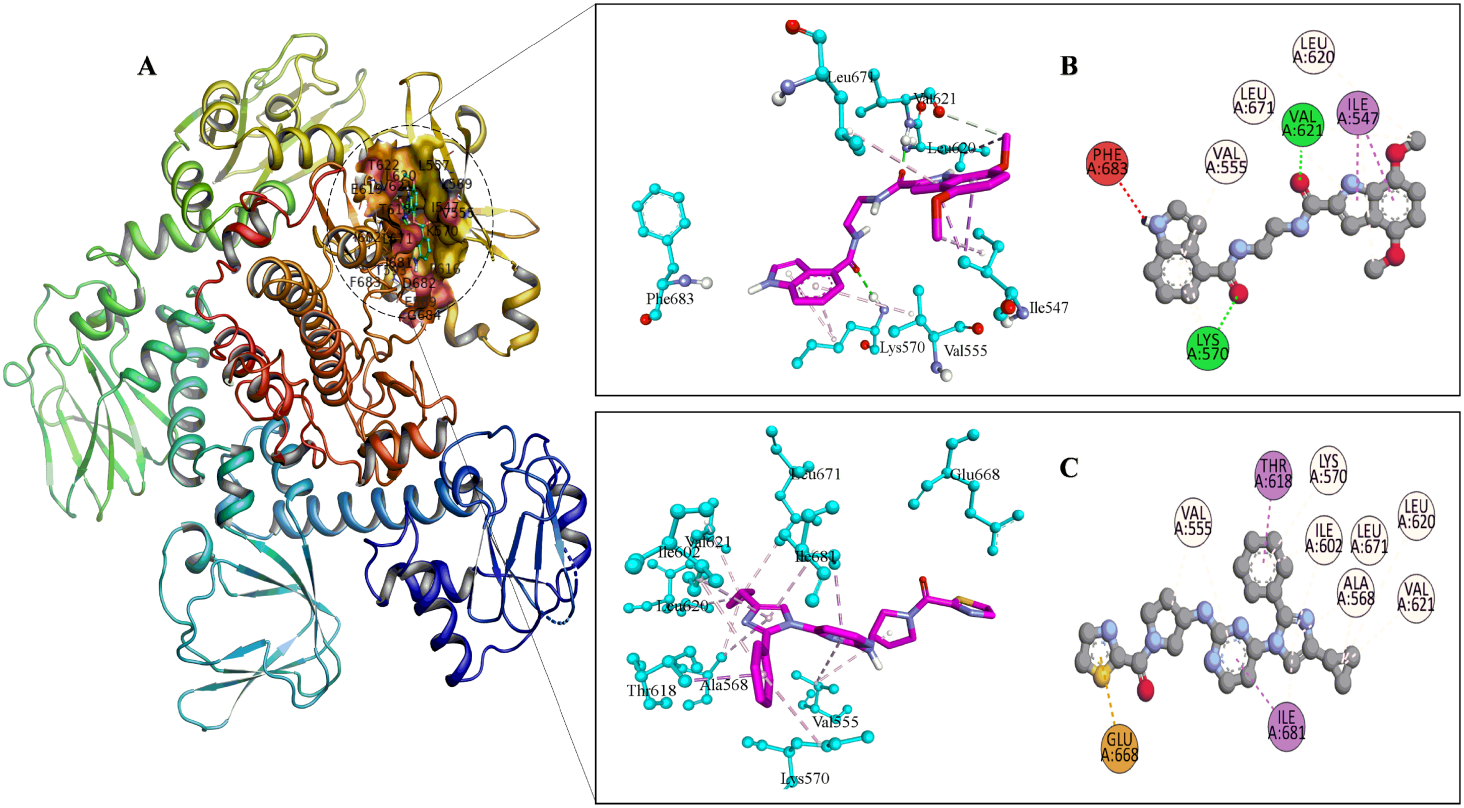
Visual representation of the site binding of Target-1 (PKG) to the reference drug and Ligand-9. **A**: 3D representation of PKG protein. **B**: PKG–Ligand-**9** 3D and 2D interaction with labeled interacting residues. **C**: PKG–reference drug 3D and 2D interaction with labeled interacting residues.

In the PKG docked complexes (**Fig 4**), the key residues involved in interactions were PHE-683, ASP-682, LEU-671, THR-622, VAL-621, LEU-620, PHE-616, ILE-602, THR-618, THR-593, LYS-570, LEU-569, LEU-557, VAL-555 and ILE-547. VAL-621 and LYS-570 contributed to strong hydrogen bonding, which enhanced structural rigidity of complex with compound 9. The pi–sigma interaction was found only in ILE-547 of the PKG-compound 9 complex and contributed to binding specificity and stabilization. ILE-681 and THR-618 stabilized the complex with reference drug by forming pi-sigma bonds. The pi–alkyl interactions that led to hydrophobic stabilization were observed at LEU-671, LEU-620 and VAL-555 in both complexes.

**Fig 5:**
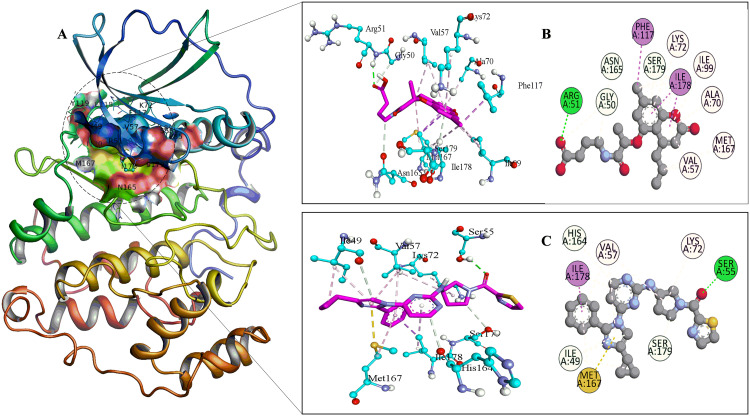
Visual representation of the site binding of Target-1 (CK2) to the reference drug and Ligand-13. **A:** 3D representation of CK2 protein. **B:** CK2–Ligand-13 3D and 2D interaction with labeled interacting residues. **C:** CK2–reference drug 3D and 2D interaction with labeled interacting residues.

In the CK2 docked complexes (**Fig 5**), the main residues involved in interactions included VAL-57, ARG-51, GLY-50, GLY-52, ASN-165, MET-167, PHE-117, SER-179, LYS-72, ILE-178, ALA-70 and ILE-99. SER-55 formed strong H-bond with CK2. Interestingly, in the CK2–Ligand-**13** docked complex, ARG-51 contributed to strong hydrogen bonding, while GLY-50 and ASN-165 participated in carbon-hydrogen bonding and SER-179 involved in pi-donor H-bond, enhancing the structural rigidity. PHE-117 and ILE-178 formed pi-sigma bond with ligand-13, while the pi-sigma interaction was found only in ILE-178 of CK2- reference drug complex contributing to the binding specificity and stabilization. In CK2-reference docked complexes, the pi–sulfur interactions were observed in MET-167. These pi–sulfur interactions resulted from the ability of sulfur atoms to engage with pi-electron clouds, which promoted hydrophobic and electrostatic complementarity between the receptor and ligand in the docked complex.

### 3.4. ADMET analysis

**Fig 6:**
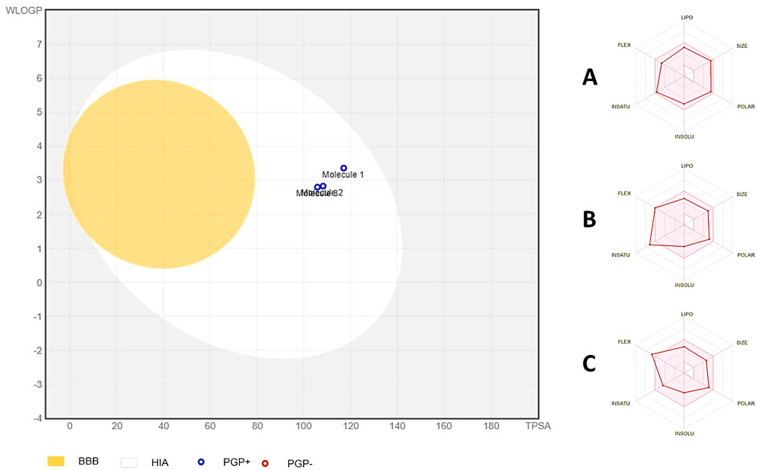
Boiled egg diagram of the reference drug and Ligand-9 and Ligand-13, in which molecule-1 refers to the reference drug, molecule-2 refers to Ligand-9, and molecule-3 represents Ligand-13. The radar charts in the left corner show A: reference drug; B: Ligand-**9**; and C: Ligand-**13**.

This study analyzed the ADME of reference drugs and lead compounds (Ligand-**9** and Ligand-**13**) and found an acceptable trend (**Table 3**). The compounds showed good gastrointestinal absorption and optimal oral bioavailability (**Fig 6**).

**Table 3:** ADME properties of the reference drug (A), Ligand-9 (B), and Ligand-13 (C).

Properties		Compound		
		A	B	C
**Physiochemical properties**	**Heavy atoms**	33	30	27
	**Aromatic heavy atoms**	22	18	10
	**Fraction Csp3**	0.29	0.18	0.45
	**No. of rotatable bonds**	7	9	10
	**No. of H-bond acceptors**	5	4	6
	**No. of H-bond donors**	1	4	2
	**MR**	130.87	113.46	102.14
	**TPSA (Å²)**	117.07	108.24	105.84
**Lipophilicity**	**iLOGP**	3.51	2.53	2.86
	**XLOGP3**	3.56	2.70	2.74
	**WLOGP**	3.36	2.83	2.80
	**MLOGP**	2.03	0.65	1.66
	**Silicos-IT logP**	3.39	3.73	1.06
	**Consensus logP**	3.17	2.49	2.83
**Solubility**	**ESOL logS**	−4.95	−3.91	−3.51
	**Ali log S**	−5.70	−4.63	−4.62
	**Silicos-IT logSw**	−6.79	−7.67	−5.90
**Pharmacokinetics**	**GI absorption**	High	High	High
	**BBB permeant**	No	No	No
	**P-gp substrate**	Yes	Yes	Yes
	**CYP1A2 inhibitor**	No	Yes	Yes
	**CYP2C19 inhibitor**	Yes	Yes	Yes
	**CYP2C9 inhibitor**	Yes	Yes	No
	**CYP2D6 inhibitor**	Yes	Yes	No
	**CYP3A4 inhibitor**	Yes	Yes	Yes
	**logKp (cm/s)**	−6.56	−6.86	−6.64
**Drug-likeness**	**Lipinski violations**	0	0	0
	**Ghose violations**	0	0	0
	**Veber violations**	0	0	0
	**Egan violations**	0	0	0
	**Bioavailability score**	0.55	0.55	0.56
**Medicinal chemistry**	**PAINS alerts**	0	0	0
	**Synthetic accessibility**	4.23	2.91	3.85

Physiochemical properties indicated that the compounds did not violate major drug development guidelines, based on Lipinski’s rule of five. All the compounds were moderately soluble, with Ligand-**13** showing the best solubility. The compounds also had moderate synthetic accessibility, with Ligand-**9** being easier to synthesize than the reference drug. However, the compounds’ enzyme inhibition properties varied slightly, with Ligand-**13** inhibiting the smallest number of enzymes, indicating lower drug interactions and efficient metabolism. Furthermore, the negative log Kp values of all compounds indicated their poor skin permeability, which can be an important consideration in their topical use. Moreover, Ghose, Veber, and Egan violations were also not observed for any compound, further strengthening their drug-likeness. There were also no PAINS alerts, which indicated the target specificity of the top-identified compounds and the reference drug.

In the toxicity analyses (**Table 4**), all three compounds showed eye irritation and corrosion. The reference drug and Ligand-**13** showed oral toxicity, but acute inhalation toxicity was not found. Ligand-**9** uniquely had a nontoxic oral toxicity prediction.

**Table 4:** StopTox toxicity parameters of the reference drug (A), Ligand-9 (B), and Ligand-13 (C).

Ligand	Toxicity				
	Inhalation	Oral	Dermal	Eye	Skin
A	NT	T	NT	T	NT
B	NT	NT	NT	T	NT
C	NT	T	NT	T	NT

Key: T, toxic; NT, nontoxic.

### 3.5. Bioactivity

**Fig 7:**
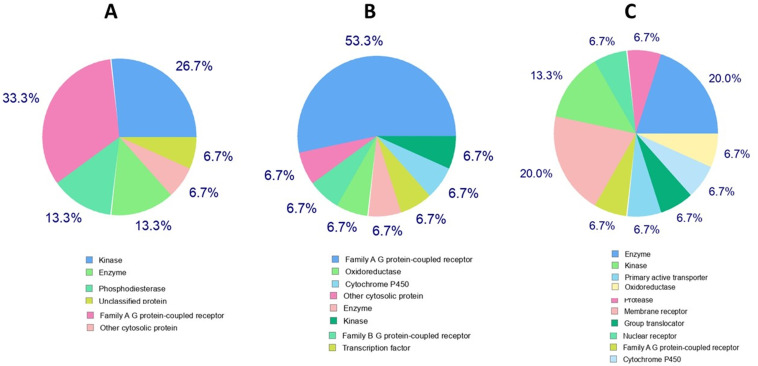
Bioactivity of the reference drug (A), Ligand-9 (B), and Ligand-13 (C).

In the bioactivity prediction, kinase, enzyme, family A-G protein-coupled receptor, and cytosolic proteins were found to be common targets of all three compounds. Ligand-**13** was found to be active against most biological targets (**Fig 7C**), indicating the diversity of its interactions. For the reference drug, the major targets were family A-G protein-coupled receptors, while kinase and enzyme proteins were secondary (**Fig 7A**). Ligand-**9** (**Fig 7B**) was also predominantly active against family A-G protein-coupled receptors, followed by oxidoreductase and other cytosolic proteins.

### 3.6. Density function theory calculations

The electronic properties and binding energies of the top-identified compounds, compared to those of the reference drug, revealed important details regarding their reactivity and stability (**Table 5**). All the compounds were predicted to be moderately reactive in terms of DFT-calculated parameters. The reference drug was found to be reactive and stable at the same time, owing to an energy gap of 4.59 eV and a high ionization potential and hardness. Its electrophilicity of 6.172 eV was comparatively moderate. Ligand-**9** showed the highest chemical reactivity due to the smallest energy gap of 3.79 eV, the highest softness score of 0.527 eV, and moderate electrophilicity (6.038 eV). In contrast, its lower ionization potential (5.283 eV) marked it as the least stable of the three compounds. Ligand-**13** was found to be intermediately reactive and stable due to an energy gap of 4.14 eV and 5.283 eV ionization potential, respectively. However, it had the highest electrophilicity (7.073 eV), electron affinity (1.759 eV), and electronegativity (3.827 eV) of all three compounds, indicating its increased reactivity due to increased capability of accepting electrons. Furthermore, it also had the highest dipole moment of 7.27 Debye, which indicated the strongest molecular polarity.

**Table 5:** Parameters of density function theory analysis of the reference drug (A), Ligand-9 (B), and Ligand-13 (C).

Property	A	B	C
**Dipole moment (Debye)**	5.1313	5.6541	7.2744
**HOMO (eV)**	−6.064	−5.283	−5.895
**LUMO (eV)**	−1.471	−1.488	−1.759
**Energy gap (ΔE gap)**	4.59	3.97	4.14
**Ionization potential (eV)**	6.064	5.283	5.895
**Electron affinity (eV)**	1.471	1.488	1.759
**Electronegativity χ (eV)**	3.767	3.385	3.827
**Electrochemical potential μ (eV)**	−3.767	−3.385	−3.827
**Hardness η (eV)**	2.296	1.897	2.068
**Softness S (eV)**	0.435	0.527	0.483
**Electrophilicity ω (eV)**	6.172	6.038	7.073

**Fig 8:**
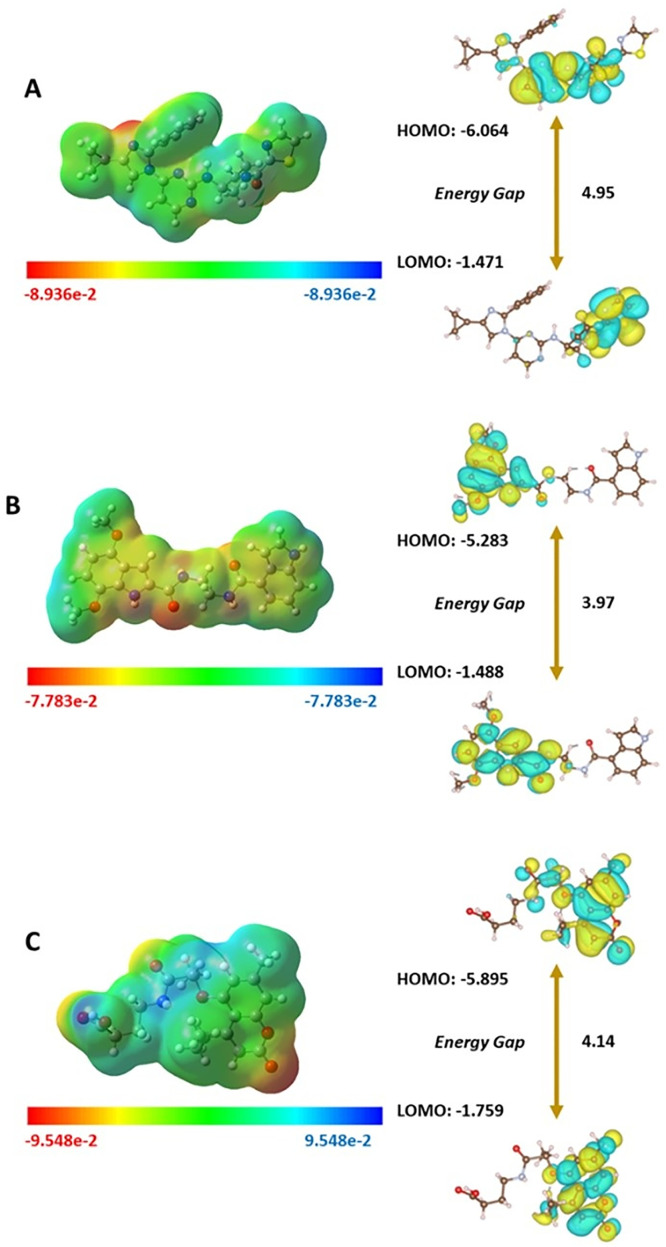
Visual representation of the molecular electrostatic potential and HOMO–LUMO energy gap of the reference drug (A), Ligand-9 (B), and Ligand-13 (C).

In addition, MEP surfaces were created to predict the regions of the selected compounds that might participate in electrophilic or nucleophilic reactions. The reference drug had an even potential distribution, while Ligand-**9** and Ligand-**13** had strong potential sites as indicated by the color-coded MEP in **Fig 8**.

Ligand-13 was just above the classification threshold in the StopTox model, with an estimated 55% chance of acute oral toxicity. Given that substances with a high resemblance to poisonous references usually score ≥70–80%, this suggests a borderline or moderate risk rather than a substantial toxic liability. Ligand-9 performed worse than Ligand-13 in crucial measures, such as the CYP enzyme inhibition profile, DFT reactivity descriptors, and docking affinity, which are crucial for drug development, even though Ligand-9 was projected to be non-toxic (51%). For MD simulation, Ligand-13 was prioritized for additional evaluation in the MD simulation.

### 3.7. Molecular dynamics simulations

**Fig 9:**
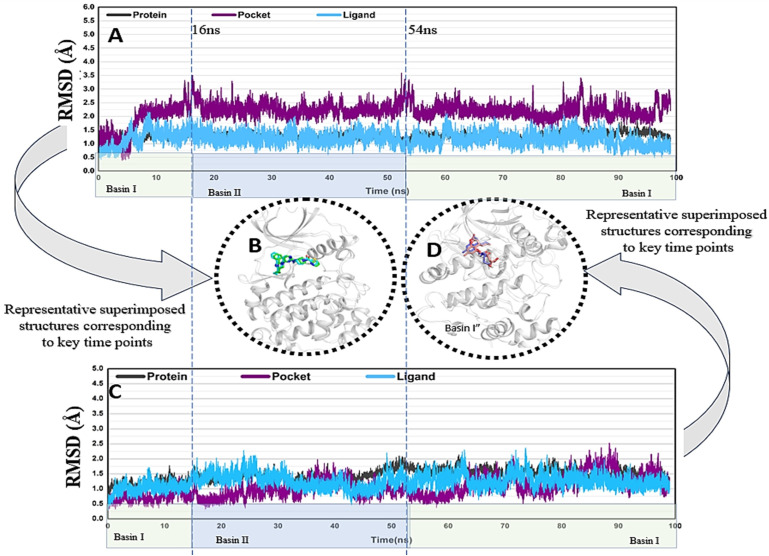
Root mean square deviation (RMSD) of the docked complex of CK2 with reference drug (A) and with Ligand-13 (C), with the highlighted superimposed structures B and D, respectively. The RMSD plot of the protein–ligand complex displayed two notable fluctuations around 16 ns and 54 ns, indicating transitions between distinct conformational states. Based on the RMSD trajectory, the system remained stable during 0–16 ns (Basin I), shifted to a new conformation from 16–54 ns (Basin II), and then returned toward the initial stable state after 54 ns (Basin I).

Ligand-**13** in complex with Target**-2 (CK2)** was narrowed down for MD simulation due to stronger binding affinity and maximum stability with most biologically active targets. For computational ease, only the actively interacting chain of the target protein (CK2α) was included in the simulations. The RMSD graphs (**Fig 9**) revealed overall stable binding of the docked complexes as indicated by a plateauing trend in the graphs. In both complexes, the target protein, their binding site, and the bound compounds, were all in close proximity. The deviations of the docked complex with the reference drug were within an acceptable range, i.e., 3.5 Å, and were prominently different around 16 ns and 54 ns. On the other hand, the docked complex with Ligand-**13** showed that all the deviations were notably different around 16 ns and 54 ns and ranged within 2.5 Å.

**Fig 10:**
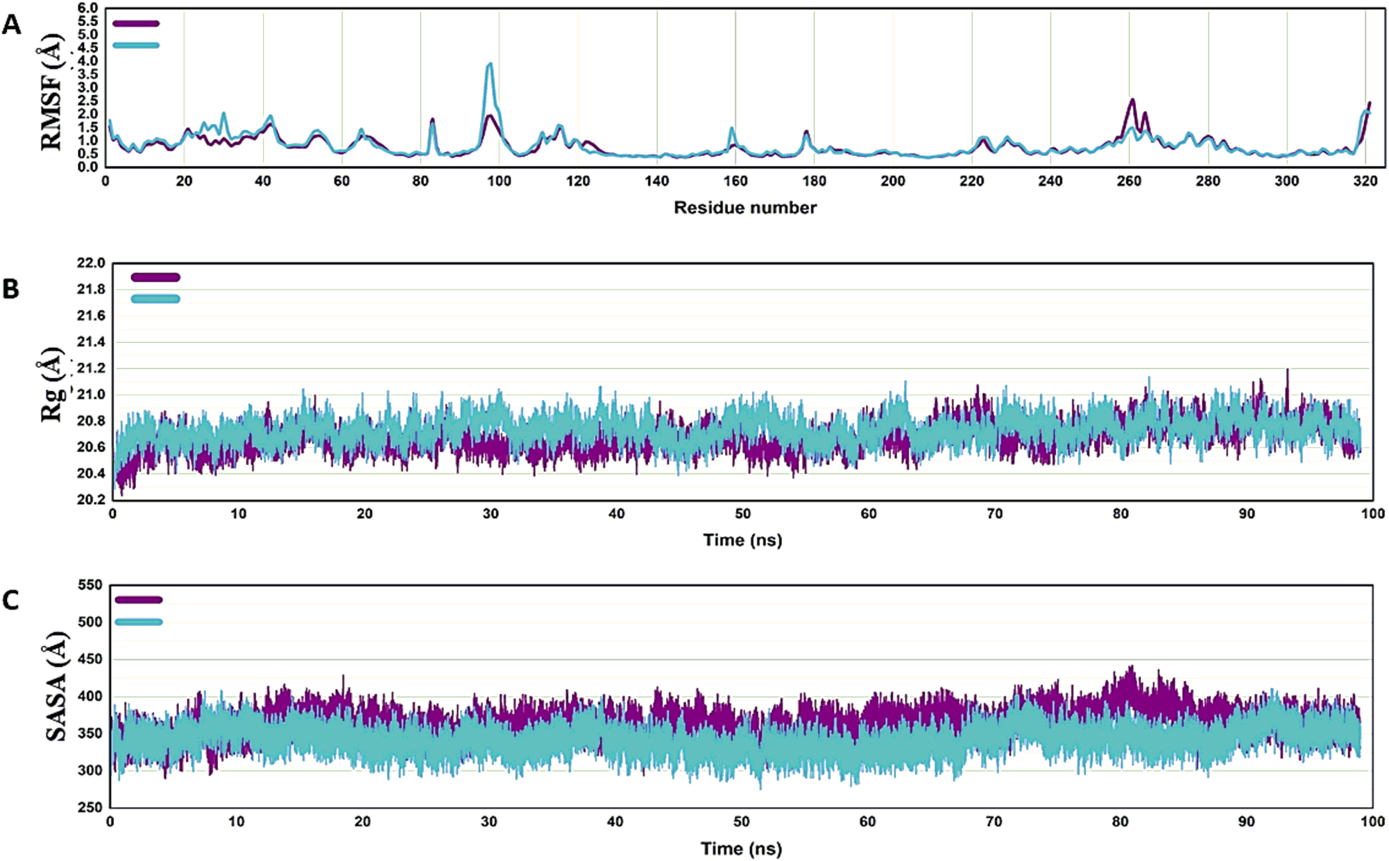
A, B, and C plots of the docked complex of CK2 with Ligand-13 (blue) and the reference drug (purple) show the root mean square fluctuation (RMSF), radius of gyration (Rg), and solvent-accessible surface area (SASA), respectively.

The RMSF (**Fig 10A**) showed a similar trend, in which slight fluctuations were observed around residue numbers 100 and 260 of the protein, which were attributed to the terminal region of the protein structure. The radius of gyration of the docked complexes was within 21.2 Å, indicating that they remained compact throughout the simulation (**Fig 10B**). The SASA varied between 400 and 450 Å, suggesting that the protein structures underwent minute conformational changes when exposed to solvents (**Fig 10C**).

**Fig 11:**
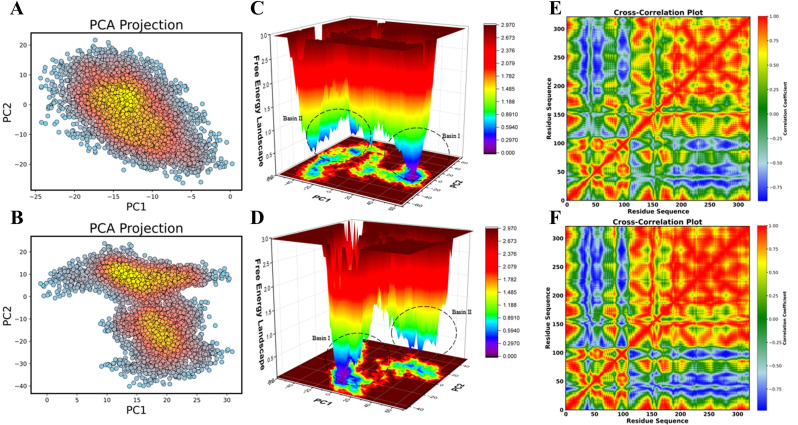
Comparative representation of principal component analysis (PCA), free energy landscape, and dynamic cross-correlation of the docked complex of CK2 with the reference drug (A, C, E) and with Ligand-13 (B, D, F).

During simulations with the reference medication, principal component analysis revealed compact clustering of the protein confirmations, indicating the stability of their docked complexes (**Fig 11A**, **11B**). However, the highly uneven distribution of the CK2-Ligand-13 complex suggests that it becomes more flexible upon binding.

The free energy landscapes (FEL) of both docked complexes (**Fig 11C**, **11D**) showed deep blue basins at various points, indicating energetically stable conformational states during ligand binding. In accordance with the conformational variability of the complexes, the large red areas were clearly indicative of higher-energy conformations. Interestingly, the FEL showed two different minima, suggesting that both complexes had two stable conformational states during the simulations. The shallower blue region, observed between 16 and 54 ns, represents a momentarily less stable state before the system returns to its original basin, whereas the deep blue region corresponds to Basin I, which is the most stable conformation observed at 0–16 and after 54 ns. The complex underwent reversible structural transformations prior to settling in an energetically advantageous state, as indicated by the consistency between the RMSD and FEL. This conclusion is further supported by the quantitative examination of basin populations and average RMSD/FEL values (**Supplementary Tables S2 and S3 in** S1 File).

Finally, the correlated and anticorrelated movements of the protein in both docked complexes were revealed by a consistent pattern inter-residue DCCM analysis (**Fig 11E**, **11F**), with positive and negative correlations indicated by red and blue colors, respectively. It was noteworthy that for the residues 0–100 (N-lobe of CK2 chain A), the negative correlations were dominant in Ligand-**13**’s complex. In contrast, Ligand-**13** exhibited increased positive correlations from residues 100–300 (C-lobe of CK2α).

**Fig 12:**
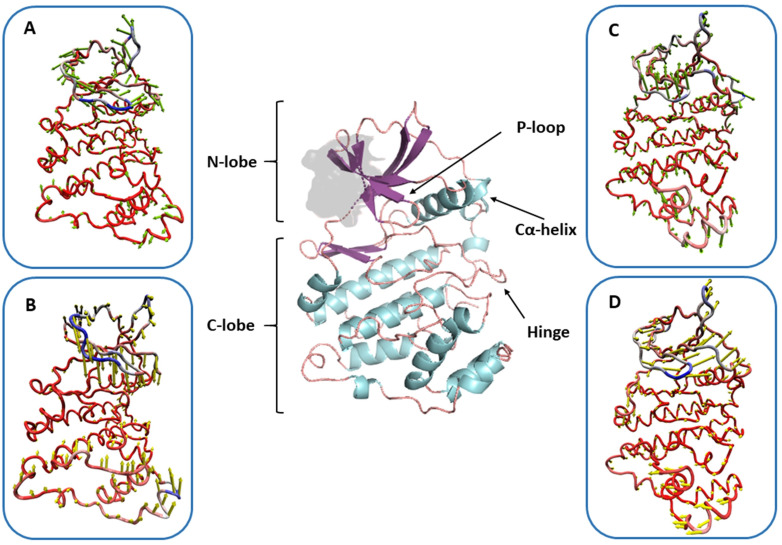
Comparative visualization of the directional movements of protein during the docking of CK2 with the reference drug (A, B) and with Ligand-13 (C, D) observed at different intervals. The detailed reference structure of the protein is presented in the center.

During simulations, the directional movements of the docked complexes in their minimum energy confirmations were also visualized (**Fig 12**). These observed movements were compared to the protein structure (CK2α). The shorter N-lobe of the protein structure, which also contained its active binding site and P-loop, was observed to constantly display movements throughout the simulation. On the other hand, the C-lobe also displayed movements, but in the opposite direction to the N-lobe. These movements were significant for the effective docking of compounds.

### 3.8. Free binding energy MMGBSA calculations

**Fig 13:**
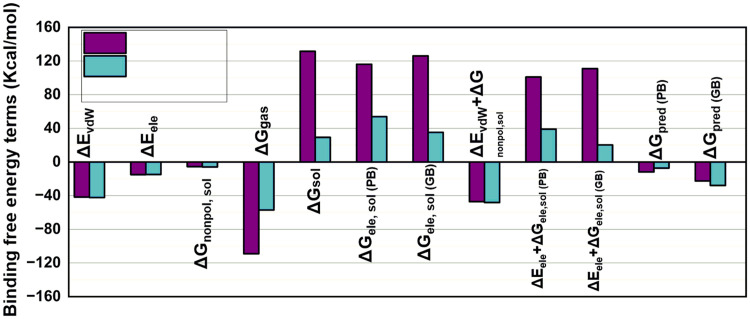
Comparative analysis of Δ*E* and Δ*G* values of the reference drug (purple) and Ligand-13 (blue).

In the comparative analysis of the docked complexes of the reference drug and Ligand-**13**, both complexes’ Δ*E* and Δ*G* showed similar results, except for a large difference in Δ*G* values in the gas and solvent phase shown in **Fig 13** (Supplementary Table S4 in S1 File). The docked complex of the reference drug exhibited more negative Δ*G*gas signaling toward its strong gas-phase interactions, which were also supported by van der Waals and electrostatic interaction energy values. On the other hand, the docked complex of Ligand-**13** displayed better binding in solvated states, as predicted by its Δ*E* and Δ*G* values*.*

## 4. Discussion

In this study, we explored the potential of naturally derived compounds as novel antimalarial agents targeting *P. falciparum*’s protein kinases, PKG and CK2, which are essential to various stages of the parasite’s life cycle [14,21]. Given the rise in resistance to antimalarial treatments, natural compounds and their derivatives are important in drug discovery. Natural materials have traditionally offered a diverse chemical pool for the development of antimalarial drugs [48]. Plants are the source of several effective antimalarial medications, such as quinine [49] and artemisinin, which form the basis of contemporary therapy. However, there has been a rise in pathogen resistance to these medications [50]. This demonstrates how the natural world may offer the next wave of treatments for malaria that can successfully combat drug resistance.

With docking scores of −7.490 kcal/mol and −11.468 kcal/mol of Ligand-9 and Ligand-13 respectively, were identified as strong inhibitors of the target proteins (PKG and CK2) in this study. The strong binding affinities of these substances, particularly Ligand-13, indicate their considerable capacity to disrupt the life cycle of *P. falciparum*. The significance of PKG and CK2 in parasite pathogenicity was confirmed by their suppression. This study expands on earlier research on the inhibition of CK2 and PKG, which have been shown to be effective therapeutic targets for the treatment of malaria [12,51].

Based on the outcomes of specific interactions, including pi–sulfur interactions, hydrogen bonding, van der Waals forces, and special salt bridges, these compounds can effectively inhibit target proteins by forming stable complexes with them. Other studies have also confirmed the inhibition of the resistance of particular *P. falciparum* through compounds with similar docking scores as observed in this study [21]. These results underscore the significance of these naturally derived products as potential candidates for the development of antimalarial drugs.

The ADME properties of Ligand-**9** and Ligand-**13** were found to be acceptable enough to consider them good antimalarial drug candidates. Gastrointestinal absorption, important for the efficacy of the drug in the human body, was found to be high for both these compounds. However, they were not predicted to cross the blood–brain barrier (BBB), which should be addressed specifically in relation to the disease and the mechanism of action of drugs. Generally, *P. falciparum* refrains from targeting brain cells. However, in cases of faulty diagnosis and improper medication, this parasite may invade brain cells, giving rise to a severe progressive form of the infection called cerebral malaria [52]. The naturally derived compounds discussed in this study are proposed to be effective for the widespread form of *P. falciparum* infections. However, they can be used against the more severe form of the disease if they are used in conjugation with other BBB-crossing antimalarial compounds [53]. The predicted bioactivity of Ligand-**9** and Ligand-**13** further reinforces the use of these compounds as antimalarials. Ligand-**13**, in particular, was found to be effective against multiple biological targets and might be highly effective in targeting the complexity of malarial infection [54].

Concerns regarding the safety of Ligand-13 and the reference compound as antimalarials are raised by their expected oral toxicity. However, toxicity is dose-dependent and can be reduced by changing the concentration or dosage of the drug [55]. Oral toxicity can be readily reduced by efficient dose adjustment of these drugs. Additionally, the distribution of medications can be modified to ensure their safe and efficient use [56]. Ligand-13’s intermediate oral toxicity prediction of 55% does not hinder its advancement in therapeutic development. Given its superior pharmacological profile and the hope that future formulations or dosing schemes would successfully reduce its mild oral toxicity signal, Ligand-13’s priority is still scientifically warranted.

For optimal drug designing, DFT-calculated parameters are essential [57]. For this purpose, the electronic properties and reactivity of Ligand-**9** and Ligand-**13,** in comparison with the reference drug, were determined. This analysis showed that Ligand**-9** with the lowest energy gap (3.79 eV) was the most reactive but it was less stable due to its lower ionization potential, as indicated by similar results in other studies [58]. However, Ligand-**13** showed optimum drug properties with an intermediate HOMO–LUMO energy gap (4.14 eV), maximum electrophilicity, and a dipole moment of 7.27 Debye that provides suitable interaction ability.

As docking is a static representation of a molecule’s binding position in the protein’s active site, MD simulations often integrate Newton’s classical equation of motion to calculate atom motions over time [59]. Molecular dynamics simulations were used to predict the ligand-binding status in a physiological environment. MD simulations of the reference drug and Ligand-**13** in complex with CK2 revealed the stable binding of protein with both these compounds. Our 100 ns MD simulation findings are consistent with earlier research that was carried out over longer timeframes (200 ns), which also demonstrated a robust and consistent interaction between the amino acids and the inhibitors of the simulation’s duration [60,61]. The conformational changes observed in the protein structure over time were similar for both docked complexes, which are predictive of similar inhibition of the target protein by Ligand-**13**. The movements observed in the N-loop of the protein signal toward flexible accommodation of Ligand-**13** in the active site are crucial for its inhibitory functions. This region is particularly significant for the functioning of CK2; its inhibition can result in the elimination of *P. falciparum* [24]. Our study reaffirms that naturally derived products, particularly Ligand-**13**, hold promise for overcoming the resistance to current therapies.

In contrast to earlier *in silico* screenings of *P. falciparum* kinases, this study combined multi-target parallel docking against PKG and CK2 with large-scale pharmacophore-based screening of a ZINC natural subset. This was followed by DFT reactivity assessment, MD-based stability analysis, and MM/GBSA binding energy profiling. This integrated workflow prioritizes Ligand-13 as a lead for additional biological assessment by reducing the number of candidates based on projected reactivity, safety liability, and binding affinity.

Although *in silico* tools highlighted useful naturally derived antimalarial compounds, these findings need to be validated via *in vitro* and *in vivo* experiments. In addition, more drug targets of *P. falciparum* can be used to combat the multi-targeted drug resistance of this deadly pathogen.

## 5. Conclusion

By assessing the activity of naturally occurring chemicals, our *in silico* method proposed Ligand-13 as a possible antimalarial medication candidate against probable *P. falciparum* targets, PKG and CK2. Comparing Ligand-13 to the other studied ligands, Ligand-13 showed the most promising combination of robust electronic characteristics, good CYP enzyme interaction profile, and greater binding affinity. Together, our results provide valuable new insights into the potential of Ligand-13 as a lead scaffold. Ligand-13 has the potential to be a lead compound, even though *in silico* models indicate a moderate probability of acute oral toxicity (55%) for this compound. Instead, it emphasizes the importance of continuing to optimize the medication development process. Future research should focus on dose-dependent toxicity assessment, alternative administration methods, and structural refinement to reduce toxicity risk. Confirming the safety profile of the compound would also require experimental validation using *in vitro* cytotoxicity and metabolic stability assays, *in vivo* acute oral range-finding, and hERG liability investigations.

## Supporting information

S1 File Supplementary data related to docking and molecular dynamics analyses. This file contains Supplementary Tables S1–S4, including the summary of interacting residues and interaction types in docked complexes, quantitative RMSD and free energy landscape (FEL) analyses from 100 ns molecular dynamics simulations of Ligand-13 and the reference drug, and energy difference values of docked complexes with target 2.
(DOCX)

## References

[1] Ntoumi F, Aklillu E, Asogun D, Ansumana R, Mfinanga S, Yeboah-Manu D, et al. Malaria resurgence in Africa: confronting the challenges. Lancet Infect Dis. 2025;25(10):1066–8. doi: 10.1016/S1473-3099(25)00499-2 40819650

[2] Talapko J, Škrlec I, Alebić T, Jukić M, Včev A. Malaria: The Past and the Present. Microorganisms. 2019;7(6):179. doi: 10.3390/microorganisms7060179 31234443 PMC6617065

[3] Venkatesan P. The 2023 WHO World malaria report. Lancet Microbe. 2024;5(3):e214. doi: 10.1016/S2666-5247(24)00016-8 38309283

[4] Maier AG, Matuschewski K, Zhang M, Rug M. Plasmodium falciparum. Trends Parasitol. 2019;35(6):481–2. doi: 10.1016/j.pt.2018.11.010 30595467

[5] Arya A, Kojom Foko LP, Chaudhry S, Sharma A, Singh V. Artemisinin-based combination therapy (ACT) and drug resistance molecular markers: A systematic review of clinical studies from two malaria endemic regions - India and sub-Saharan Africa. Int J Parasitol Drugs Drug Resist. 2021;15:43–56. doi: 10.1016/j.ijpddr.2020.11.006 33556786 PMC7887327

[6] Maiga FO, Wele M, Toure SM, Keita M, Tangara CO, Refeld RR, et al. Artemisinin-based combination therapy for uncomplicated Plasmodium falciparum malaria in Mali: a systematic review and meta-analysis. Malar J. 2021;20(1):356. doi: 10.1186/s12936-021-03890-0 34461901 PMC8404312

[7] Woodrow CJ, White NJ. The clinical impact of artemisinin resistance in Southeast Asia and the potential for future spread. FEMS Microbiol Rev. 2017;41(1):34–48. doi: 10.1093/femsre/fuw037 27613271 PMC5424521

[8] Hanboonkunupakarn B, White NJ. Advances and roadblocks in the treatment of malaria. Br J Clin Pharmacol. 2022;88(2):374–82. doi: 10.1111/bcp.14474 32656850 PMC9437935

[9] Meibalan E, Marti M. Biology of malaria transmission. Cold Spring Harbor Perspectives in Medicine. 2017;7(3). 10.1101/cshperspect.a025452 PMC5334247 27836912

[10] Taylor HM, McRobert L, Grainger M, Sicard A, Dluzewski AR, Hopp CS, et al. The malaria parasite cyclic GMP-dependent protein kinase plays a central role in blood-stage schizogony. Eukaryot Cell. 2010;9(1):37–45. doi: 10.1128/EC.00186-09 19915077 PMC2805293

[11] Rotella D, Siekierka J, Bhanot P. Plasmodium falciparum cGMP-Dependent Protein Kinase - A Novel Chemotherapeutic Target. Frontiers in Microbiology. 2020;11:610408. 33613463 10.3389/fmicb.2020.610408 PMC7886688

[12] Baker DA, Matralis AN, Osborne SA, Large JM, Penzo M. Targeting the Malaria Parasite cGMP-Dependent Protein Kinase to Develop New Drugs. Front Microbiol. 2020;11:602803. doi: 10.3389/fmicb.2020.602803 33391223 PMC7773720

[13] Dastidar EG, Dayer G, Holland ZM, Dorin-Semblat D, Claes A, Chêne A, et al. Involvement of Plasmodium falciparum protein kinase CK2 in the chromatin assembly pathway. BMC Biol. 2012;10:5. doi: 10.1186/1741-7007-10-5 22293287 PMC3296614

[14] Hitz E, Grüninger O, Passecker A, Wyss M, Scheurer C, Wittlin S, et al. The catalytic subunit of Plasmodium falciparum casein kinase 2 is essential for gametocytogenesis. Commun Biol. 2021;4(1):336. doi: 10.1038/s42003-021-01873-0 33712726 PMC7954856

[15] Bheemanaboina RRY, de Souza ML, Gonzalez ML, Mahmood SU, Eck T, Kreiss T, et al. Discovery of Imidazole-Based Inhibitors of Plasmodium falciparum cGMP-Dependent Protein Kinase. ACS Med Chem Lett. 2021;12(12):1962–7. doi: 10.1021/acsmedchemlett.1c00540 34917261 PMC8667308

[16] Gilleran JA, Ashraf K, Delvillar M, Eck T, Fondekar R, Miller EB, et al. Structure-Activity Relationship of a Pyrrole Based Series of PfPKG Inhibitors as Anti-Malarials. J Med Chem. 2024;67(5):3467–503. doi: 10.1021/acs.jmedchem.3c01795 38372781

[17] Tajuddeen N, Van Heerden FR. Antiplasmodial natural products: an update. Malar J. 2019;18(1):404. doi: 10.1186/s12936-019-3026-1 31805944 PMC6896759

[18] Siddiqui A, Iram F, Siddiqui S, Sahu K. Role of natural products in drug discovery process. Int J Drug Dev Res. 2014;6:172–204.

[19] Abdullahi SH, Uzairu A, Shallangwa GA, Uba S, Umar AB. In-silico activity prediction, structure-based drug design, molecular docking and pharmacokinetic studies of selected quinazoline derivatives for their antiproliferative activity against triple negative breast cancer (MDA-MB231) cell line. Bull Natl Res Cent. 2022;46(1). doi: 10.1186/s42269-021-00690-z

[20] Ahmed AH, Alkali YI. In silico Pharmacokinetics and Molecular Docking Studies of Lead Compounds Derived from Diospyros Mespiliformis. PharmaTutor. 2019;7(3):31. doi: 10.29161/pt.v7.i3.2019.31

[21] Vanaerschot M, Murithi JM, Pasaje CFA, Ghidelli-Disse S, Dwomoh L, Bird M, et al. Inhibition of Resistance-Refractory P. falciparum Kinase PKG Delivers Prophylactic, Blood Stage, and Transmission-Blocking Antiplasmodial Activity. Cell Chem Biol. 2020;27(7):806–816.e8. doi: 10.1016/j.chembiol.2020.04.001 32359426 PMC7369637

[22] Burley SK, Berman HM, Kleywegt GJ, Markley JL, Nakamura H, Velankar S. Protein Data Bank (PDB): the single global macromolecular structure archive. Protein crystallography: methods and protocols. 2017:627–41. 10.1007/978-1-4939-7000-1_26 PMC5823500 28573592

[23] Ruiz-Carrillo D, Lin J, El Sahili A, Wei M, Sze SK, Cheung PCF, et al. The protein kinase CK2 catalytic domain from Plasmodium falciparum: crystal structure, tyrosine kinase activity and inhibition. Sci Rep. 2018;8(1):7365. doi: 10.1038/s41598-018-25738-5 29743645 PMC5943518

[24] Koes DR. The Pharmit Backend: A Computer Systems Approach to Enabling Interactive Online Drug Discovery. IBM J Res Dev. 2018;62(6):1–6. doi: 10.1147/jrd.2018.2883977 33871478 PMC8049614

[25] Irwin JJ, Shoichet BK. ZINC--a free database of commercially available compounds for virtual screening. J Chem Inf Model. 2005;45(1):177–82. doi: 10.1021/ci049714+ 15667143 PMC1360656

[26] O’Boyle NM, Banck M, James CA, Morley C, Vandermeersch T, Hutchison GR. Open Babel: An open chemical toolbox. J Cheminform. 2011;3:33. doi: 10.1186/1758-2946-3-33 21982300 PMC3198950

[27] Manzoor H, Khan MU, Khan S, Shah M, Shabbir CA, Alkhtani HM. Linalool-based silver nanoconjugates as potential therapeutics for glioblastoma: in silico and in vitro insights. PLoS One. 2025;20(6):e0325281. doi: 10.1371/journal.pone.0325281 40504869 PMC12161535

[28] Nautiyal M, Sekaran K, Sekaran S, Rengasamy G, Veeraraghavan VP, Eswaramoorthy R. Molecular docking analysis of Indole based diaza-sulphonamides with JAK-3 protein. Bioinformation. 2023;19(1):74–8. doi: 10.6026/97320630019074 37720295 PMC10504512

[29] Eberhardt J, Santos-Martins D, Tillack AF, Forli S. AutoDock Vina 1.2.0: New docking methods, expanded force field, and Python bindings. J Chem Inf Model. 2021;61(8):3891–8. 34278794 10.1021/acs.jcim.1c00203 PMC10683950

[30] Zaelani BF, Safithri M, Andrianto D. Molecular docking of red betel (Piper crocatum Ruiz & Pav) bioactive compounds as HMG-CoA reductase inhibitor. Journal of Scientific and Applied Chemistry. 2021;24(3):101–7.

[31] Oum YH, Kell SA, Yoon Y, Liang Z, Burger P, Shim H. Discovery of novel aminopiperidinyl amide CXCR4 modulators through virtual screening and rational drug design. Eur J Med Chem. 2020;201:112479. doi: 10.1016/j.ejmech.2020.112479 32534343 PMC7422936

[32] Yuan S, Chan HCS, Hu Z. Using PyMOL as a platform for computational drug design. WIREs Comput Mol Sci. 2017;7(2). doi: 10.1002/wcms.1298

[33] Jejurikar BL, Rohane SH. Drug designing in discovery studio. 2021.

[34] Sakhawat A, Khan MU, Rehman R, Khan S, Shan MA, Batool A, et al. Natural compound targeting BDNF V66M variant: insights from in silico docking and molecular analysis. AMB Express. 2023;13(1):134. doi: 10.1186/s13568-023-01640-w 38015338 PMC10684480

[35] Daina A, Michielin O, Zoete V. SwissADME: a free web tool to evaluate pharmacokinetics, drug-likeness and medicinal chemistry friendliness of small molecules. Sci Rep. 2017;7:42717. doi: 10.1038/srep42717 28256516 PMC5335600

[36] Borba JVB, Alves VM, Braga RC, Korn DR, Overdahl K, Silva AC, et al. STopTox: An in Silico Alternative to Animal Testing for Acute Systemic and Topical Toxicity. Environ Health Perspect. 2022;130(2):27012. doi: 10.1289/EHP9341 35192406 PMC8863177

[37] Daina A, Michielin O, Zoete V. SwissTargetPrediction: updated data and new features for efficient prediction of protein targets of small molecules. Nucleic Acids Res. 2019;47(W1):W357–64. doi: 10.1093/nar/gkz382 31106366 PMC6602486

[38] Frisch M. Gaussian 09, Revision D. 01. Gaussian, Inc. 2009.

[39] Momma K, Izumi F. VESTA: a three-dimensional visualization system for electronic and structural analysis. J Appl Crystallogr. 2008;41(3):653–8. doi: 10.1107/s0021889808012016

[40] Manzoor H, Khan MU, Khan S, Haider N, Ullah MI, Ghanem HB, et al. Citronellol silver nanoconjugates as a therapeutic strategy for glioblastoma through computational and experimental evaluation. Sci Rep. 2025;15(1):34076. doi: 10.1038/s41598-025-14557-0 41028248 PMC12484693

[41] Jabeen S, Khan MU, Ejaz H, Waqar S, Farhana A, Alruwaili M, et al. Identifying novel inhibitors against drug-resistant mutant CYP-51 Candida albicans: A computational study to combat fungal infections. PLoS One. 2025;20(3):e0318539. doi: 10.1371/journal.pone.0318539 40036223 PMC11878927

[42] Arantes PR, Polêto MD. Making it rain: cloud-based molecular simulations for everyone. J Chem Inf Model. 2021;61(10):4852–6. 34595915 10.1021/acs.jcim.1c00998

[43] Grant BJ, Skjaerven L, Yao X-Q. The Bio3D packages for structural bioinformatics. Protein Sci. 2021;30(1):20–30. doi: 10.1002/pro.3923 32734663 PMC7737766

[44] Palma J, Pierdominici-Sottile G. On the Uses of PCA to Characterise Molecular Dynamics Simulations of Biological Macromolecules: Basics and Tips for an Effective Use. Chemphyschem. 2023;24(2):e202200491. doi: 10.1002/cphc.202200491 36285677

[45] Kitao A. Principal component analysis and related methods for investigating the dynamics of biological macromolecules. J. 2022;5(2):298–317.

[46] Chen S-F, Cao Y, Chen J-J, Chen J-Z. Binding selectivity studies of PKBα using molecular dynamics simulation and free energy calculations. J Mol Model. 2013;19(11):5097–112. doi: 10.1007/s00894-013-1997-3 24085537

[47] Qian H, Chen J, Pan Y, Chen J. Molecular Modeling Studies of 11β-Hydroxysteroid Dehydrogenase Type 1 Inhibitors through Receptor-Based 3D-QSAR and Molecular Dynamics Simulations. Molecules. 2016;21(9). 10.3390/molecules21091222 PMC6274164 27657020

[48] Nain T, Sharma S, Chawariya N, Yadav JP. Prospect of natural compounds against malaria: a review. Bulletin of Pharmaceutical Sciences Assiut University. 2022;45(2):629–53.

[49] Tisnerat C, Dassonville-Klimpt A, Gosselet F, Sonnet P. Antimalarial Drug Discovery: From Quinine to the Most Recent Promising Clinical Drug Candidates. Curr Med Chem. 2022;29(19):3326–65. doi: 10.2174/0929867328666210803152419 34344287

[50] Lu F, Culleton R, Zhang M, Ramaprasad A, von Seidlein L, Zhou H, et al. Emergence of Indigenous Artemisinin-Resistant Plasmodium falciparum in Africa. N Engl J Med. 2017;376(10):991–3. doi: 10.1056/NEJMc1612765 28225668

[51] Tomaz KCP, Tavella TA, Borba JVB, Salazar-Alvarez LC, Levandoski JE, Mottin M, et al. Identification of potential inhibitors of casein kinase 2 alpha of Plasmodium falciparum with potent in vitro activity. Antimicrob Agents Chemother. 2023;67(11):e0058923. doi: 10.1128/aac.00589-23 37819090 PMC10649021

[52] Panda C, Mahapatra RK. An update on cerebral malaria for therapeutic intervention. Mol Biol Rep. 2022;49(11):10579–91. doi: 10.1007/s11033-022-07625-5 35670928

[53] Mukherjee S, Ray G, Saha B, Kar SK. Oral Therapy Using a Combination of Nanotized Antimalarials and Immunomodulatory Molecules Reduces Inflammation and Prevents Parasite Induced Pathology in the Brain and Spleen of P. berghei ANKA Infected C57BL/6 Mice. Front Immunol. 2022;12:819469. doi: 10.3389/fimmu.2021.819469 35095923 PMC8793777

[54] Feldmann C, Yonchev D, Bajorath J. Structured data sets of compounds with multi-target and corresponding single-target activity from biological assays. Future Sci OA. 2021;7(5):FSO685. doi: 10.2144/fsoa-2020-0209 34046190 PMC8147869

[55] Shaban E, Salaheldin K, El sayed E, Abd El-Aziz M, Nasr S, M Desouky H, et al. Evaluation of acute oral toxicity of zinc oxide nanoparticles in rats. Egypt J Chem. 2021. doi: 10.21608/ejchem.2021.80810.4003

[56] Fourie Zirkelbach J, Shah M, Vallejo J, Cheng J, Ayyoub A, Liu J, et al. Improving Dose-Optimization Processes Used in Oncology Drug Development to Minimize Toxicity and Maximize Benefit to Patients. J Clin Oncol. 2022;40(30):3489–500. doi: 10.1200/JCO.22.00371 36095296

[57] Flores MC, Márquez EA, Mora JR. Molecular modeling studies of bromopyrrole alkaloids as potential antimalarial compounds: a DFT approach. Med Chem Res. 2017;27(3):844–56. doi: 10.1007/s00044-017-2107-3

[58] Miar M, Shiroudi A, Pourshamsian K, Oliaey AR, Hatamjafari F. Theoretical investigations on the HOMO–LUMO gap and global reactivity descriptor studies, natural bond orbital, and nucleus-independent chemical shifts analyses of 3-phenylbenzo[d]thiazole-2(3H)-imine and its para-substituted derivatives: Solvent and substituent effects. Journal of Chemical Research. 2020;45(1–2):147–58.

[59] Owoloye AJ, Ligali FC, Enejoh OA, Musa AZ, Aina O, Idowu ET, et al. Molecular docking, simulation and binding free energy analysis of small molecules as PfHT1 inhibitors. PLoS One. 2022;17(8):e0268269. doi: 10.1371/journal.pone.0268269 36026508 PMC9417013

[60] Kamunya SG. Design of Selective Plasmodium falciparum Kinase Inhibitors using Computer-Aided Drug Discovery. 2022.

[61] Kankinou SG, Yildiz M, Kocak A. Exploring potential Plasmodium kinase inhibitors: a combined docking, MD and QSAR studies. J Biomol Struct Dyn. 2024;42(17):8958–68. doi: 10.1080/07391102.2023.2249111 37599462

