# Supplementary material for: Computational screening of natural inhibitors against Plasmodium falciparum kinases: Toward novel antimalarial therapies
Source: PLoS One. 2026 Jan 13;21(1):e0339317. doi: 10.1371/journal.pone.0339317 (PMC12798981; doi:10.1371/journal.pone.0339317)
Supplement: S1 File — This file contains Supplementary Tables S1–S4, including the summary of interacting residues and interaction types in docked complexes, quantitative RMSD and free energy landscape (FEL) analyses from 100 ns molecular dynamics simulations of Ligand-13 and the reference drug, and energy difference values of docked complexes with target 2. (DOCX) [file pone.0339317.s001.docx]

**Supplementary Data**

**Supplementary Table S1.** Summary of interacting residues and type of interaction in docked complexes. A: target **1**- reference drug. B: target **1**-ligand **9**. C: target **2**-referance drug. D: target **2**- ligand **13**.

| Type of interaction | Interacting residues | | | |
| --- | --- | --- | --- | --- |
|  | **A** | **B** | **C** | **D** |
| Van der Waals | LEU 557  GLY 624  LEU 620  THR 622  ILE 602  LEU 604  PHE 616  ASP 682  GLY 548  LEU 569  THR 618 | ASP 682  PHE 683  GLU 589  THR 593  THR 618  ILE 602  VAL 555  GLU 619  ALA 568  LUE 620  THR 622 | ASN 66 (B)  CYS 65 (B)  TYR 54  LYS 162  SER 55  GLY 52  SER 179  GLY 50  PHE 117  ILE 99  ALA 70  ILE 120  TYR 119  ASN 122 | GLU 64 (B)  GLU 118  ILE 99  ASN 165  HID 164  SER 179  ASN 122  GLY 50  GLY 52  ARG 51  LYS 53  GLU 85  GLY 181  LEU 182 |
| Conventional hydrogen bond | VAL 621 | VAL 621  GLY 624 | LYS 81 | ILE 120  LYS 72 |
| Carbon hydrogen bond | GLU 619 | GLU 625  ILE 547 | GLU 64 (B)  ASN 165  HID 164 | Ser 55  Tyr 119 |
| Pi-cation | ARG 545 |  |  |  |
| Pi-sulfur |  |  | MET 167 (A) | MET 167 |
| Pi-alkyl | ILE 681  ILE 547  LEU 671  ALA 568  VAL 555  LYS 570 | LYS 570  ILE 681  LEU 671 | LYS 53  LEU 182  ILE 178  LYS 72  VAL57  ILE 49  ALL IN A |  |
| Salt bridge |  |  |  | LYS 72  LYS 81 |

**Supplementary Table S2.** Quantitative analysis of RMSD and FEL parameters showing mean and standard deviation values for each basin during the 100 ns MD simulation of the Ligand-13.

| **Basin** | **Time Range (ns)** | **RMSD_mean** | **RMSD_std** | **PC1_mean** | **PC1_std** | **PC2_mean** | **PC2_std** | **% Frames** |
| --- | --- | --- | --- | --- | --- | --- | --- | --- |
| Basin I | 0.1-15.9 | 0.989535 | 0.112565 | 17.858133 | 3.598055 | -23.514599 | 8.542258 | 15.983197 |
| Basin II | 16.0-53.9 | 1.112393 | 0.091819 | 19.225765 | 3.753335 | -12.341683 | 8.429366 | 38.007602 |
| Basin III | 54.0-100.0 | 1.155802 | 0.108043 | 15.372881 | 5.865688 | 9.529448 | 3.845722 | 46.009202 |

**Supplementary Table S3.** Quantitative analysis of RMSD and FEL parameters showing mean and standard deviation values for each basin during the 100 ns MD simulation of the Reference drug.

| **Basin** | **Time Range (ns)** | **RMSD_mean** | **RMSD_std** | **PC1_mean** | **PC1_std** | **PC2_mean** | **PC2_std** | **% Frames** |
| --- | --- | --- | --- | --- | --- | --- | --- | --- |
| Basin I | 0.1-15.9 | 0.989382 | 0.079556 | -7.752504 | 2.944137 | -10.843473 | 5.510891 | 15.983197 |
| Basin II | 16.0-53.9 | 1.134197 | 0.101077 | -14.068169 | 3.975691 | -3.688819 | 9.563558 | 38.007602 |
| Basin III | 54.0-100.0 | 1.194926 | 0.089196 | -13.432682 | 3.475590 | 1.645083 | 7.350342 | 46.009202 |

**Supplementary Table S4.** The energy difference values of the docked complex of target **2** with reference drug (A) and ligand-**13** (B).

| Parameters (kcal/mol) | A | B |
| --- | --- | --- |
| Δ*E_vdW_ ^a^* | -41.6479 | -42.1938 |
| Δ*E*_ele_ *^a^* | -15.0627 | -14.8949 |
| Δ*G*_nonpol, sol_ *^a^* | -5.5203 | -5.9306 |
| Δggas | -108.98 | -57.0887 |
| ΔG_sol_ | 131.5433 | 29.233 |
| Δ*G*_ele, sol (PB)_ *^a^* | 116.093 | 53.8786 |
| Δ*G*_ele, sol (GB)_ *^a^* | 126.023 | 35.1636 |
| Δ*E_vdW_*+Δ*G*_nonpol,sol_ *^a^* | -47.1682 | -48.1244 |
| Δ*E*_ele_+Δ*G*_ele,sol (PB)_ *^a^* | 101.0303 | 38.9837 |
| Δ*E*_ele_+Δ*G*_ele,sol (GB)_ *^a^* | 110.9603 | 20.2687 |
| Δ*G*_pred (PB)_*^b^* | -11.8685 | -7.2928 |
| Δ*G*_pred (GB)_*^b^* | -22.5633 | -27.8557 |
